# Sequences of vocational rehabilitation services in Germany: a cohort study

**DOI:** 10.1186/s12913-023-10499-3

**Published:** 2024-01-15

**Authors:** Annika Sternberg, David Fauser, Hannes Banaschak, Matthias Bethge

**Affiliations:** Institute for Social Medicine and Epidemiology, University of Lübeck, Ratzeburger Allee 160, 23562 Lübeck, Germany

**Keywords:** Vocational rehabilitation, Rehabilitation trajectories, Sequence analysis, Vocational reintegration

## Abstract

**Background:**

This study aimed to describe sequences of vocational rehabilitation services among individuals with approved vocational rehabilitation in Germany and to identify typical service sequences.

**Methods:**

We used administrative data on vocational rehabilitation services and questionnaire data on health and work ability to describe frequencies and sequences of vocational rehabilitation services financed by the Federal German Pension Insurance. Through sequence analysis, we were able to map the service sequences. We did cluster analyses to identify typical different service sequences.

**Results:**

Our sample included 1,652 individuals with 2,584 services. Integration services and two-year vocational retraining were the most common services. We could identify three different service clusters around integration services: shorter ones, followed by employer benefits and without employer benefits. We found two different clusters around two-year vocational retraining: shorter and longer clusters. Two-year vocational retraining was more often initiated by preparatory services and followed by employer benefits than integration services. Longer services in both clusters were associated with better baseline data for physical health, work ability, risk of future work disability, and younger age than shorter services. People in two-year-vocational retraining reported at baseline better general health, better work ability, low risk of future work disability, and less mental illness compared to people in integration services.

**Conclusions:**

Multiple services, that is, sequences of services, were more likely to occur among individuals with more complex services like two-year vocational retraining. Utilization of complex services and longer services was influenced by health, age, risk of future work disability, and education.

**Trial registration:**

German Clinical Trials Register DRKS00009910, registration 25/01/2016.

## Background

Chronic illness and disability affect a large and growing number of people worldwide [1] including in Germany [2]. People of working age with a chronic condition are significantly less likely to be employed than people without a chronic condition [3, 4]. In addition to the risk of poverty in old age due to reduced pension entitlements [4], the lack of labor force participation can be an additional burden that may have a negative impact on the health of those affected [5–7]. In 2021, 1.8 million people in Germany received a disability pension due to chronic illness [8]. In order to help people to return to work and to avoid disability pensions, measures to promote the return to work are of central interest to health and pension insurances in Germany.

Vocational rehabilitation (VR) services are a strategy for people with health-related reduction of work ability to return to work. The services can either aim to restore the work ability to stay in a job that is still available or to reintegrate people into working life after a period of unemployment. The understanding and implementation of VR differ internationally [9–11], but there is agreement that VR is a process that optimizes work participation [12]. It is often an interdisciplinary intervention, provided by a multidisciplinary team that collaborates with patients using the biopsychosocial model [13].

One main provider for VR services in Germany is the Federal German Pension Insurance. Together with the Federal Employment Agency, the Federal German Pension Insurance is one of the two largest providers of VR in Germany [14]. The Federal German Pension Insurance is responsible for rehabilitation services if someone has been employed for at least 15 years or is already receiving a disability pension due to health reasons. The range of available VR services is broad. There are services that aim at maintaining an existing job, such as technical working aids or an adjustment of the workplace. Other services may be directed towards obtaining a new job better fitting a person’s impairment. Services like vocational retraining are intended to expand and strengthen work-related knowledge and skills of the individuals and thus enable them to enter a new job. There are also services that provide financial support for employers to improve conditions for return to work or services that facilitate the performance of the existing work.

Participation in VR requires a claim for rehabilitation. When approving a claim, the Federal German Pension Insurance recognizes the need for VR and declares its financial responsibility, without specifying concrete rehabilitation services. One or several different rehabilitation services are selected after approval during a consultation with a rehabilitation counselor of the Federal German Pension Insurance. Further services may be approved after the initial approval. The initial approval of VR can therefore be understood as the starting point of a complex VR process or a sequence of services. Frequent access to vocational rehabilitation is via medical rehabilitation. In medical rehabilitation, occupational problems can be identified and, if necessary, already dealt with. In addition, further occupational support needs and need of VR following medical rehabilitation can be clarified and prepared.

In 2020, 365,525 applications of VR were submitted at the Federal German Pension Insurance and 248,772 approvals were made. The Federal German Pension Insurance reports that 67% of applications were approved. In the same year 125,187 services were completed. The services associated with an application are not always completed in the same year since the services can last for several months. Men completed about twice as many services as women. The most common services among men and women were services including working aids; financial support for the procurement, equipping, and maintenance of a handicapped-suitable apartment or motor vehicle; financial support for travel costs; or job-related movement (women 34%; men 56% of completed services). Women had more vocational training services (further vocational training or vocational qualification) than men (women 27%; men 17% of completed services) while men had more services that also involved maintaining an existing job [15].

A person may receive a one-time service type or different types of services or repetitive same service types. The time points of starting services, number of services, duration, type, and combination of different services result in a unique service sequence.

Although VR processes can consist of a service sequence, studies in Germany so far have often only considered single service types when assessing the success of the entire process of VR, usually long and high-cost services such as vocational retraining [16, 17]. Little is known about the service sequences and their characteristics. However, vocational reintegration is not only determined by the fact whether a service has been provided or not, but is also constructed from the type, duration, and sequence of rehabilitation services. Findings on successful vocational reintegration can thus also be supplemented by individual sequence histories. There are few descriptions of the VR service sequences so far that indicate a complex and long-lasting service sequence history [18, 19].

Several studies have described the return to work process after VR services using sequence analysis techniques to map the stages of return to work [20–23]. Sequence analysis can identify typical states of VR [24] and considers the processual nature of vocational reintegration and changes within this process [20]. Through sequence analysis, start and duration of services, service types, states of no services, and changes between states can be traced and described. The descriptions can help to identify how the VR process looks like in detail, which services take place in which order and duration, which service sequences are typical in which groups of participants, and when and where there are possible interruptions or discontinuations of the VR process.

In our study, we examined VR services among individuals with approved VR in Germany. The purpose of our study was to describe the frequency of different types of VR services, explore the individual sequences of VR services, define the main services for each service sequence, identify common service sequences, and describe the participants in these groups of common service sequences.

## Methods

### Study design and sample

Our cohort study monitored courses of VR over four years in individuals who had applied for VR by the Federal German Pension Insurance and whose VR was approved between January and June 2016. The Federal German Pension Insurance is one of the largest providers of VR services in Germany. Persons for whom the Federal German Pension Insurance is responsible for VR services differ from those of other VR providers such as regional pension insurance providers, the German Social Accident Insurance Institution or the Federal Employment Agency. For example, persons belonging to the Federal German Pension Insurance are older, have lower education and are more often employed at time of the approval of VR service than persons belonging to the Federal Employment Agency [14]. In addition, significantly more women are insured at the Federal German Pension Insurance than at the regional pension insurances. Study participants answered a questionnaire about health and work ability up to four weeks after the approval of the rehabilitation request. Administrative data on VR services between 2016 and 2020 were provided by the Federal German Pension Insurance for participants with informed consent. Study participants did not complete a VR or had any pension claims or benefits in the two years before entering our study. People were excluded from the analyses if they did not receive any services after being approved.

Our study was approved by the ethics committee of the University of Lübeck (15–374) and registered in the German Clinical Trials Register (DRKS00009910, registration 25/01/2016). The manuscript preparation followed the recommendations of the Strengthening the Reporting of Observational Studies in Epidemiology (STROBE) statement for cohort studies [25].

### Data on vocational rehabilitation services

Data on VR services included information on rehabilitation services (type, start date, end date) for the study population from January 2016 to April 2020 (52 months). The observation period was set at an average of 4 years in order to be able to map the entire range of services and also longer services (with a duration of approx. 2 years), including the time up to the start of the service and a one-year follow-up. The 52 months cover the period from the first approval to the completion of the last service of our study sample. We presented the monthly states of services or no service for each participant. In our study, we defined a sequence as the individual chronological order of services within 52 months. The sequences consisted of at least one month with a service and could include different service types or only one service type. VR services were grouped into six service categories: two-year vocational retraining, one-year vocational retraining, integration service, preparatory services, employer benefits and other services. In addition, there was the monthly state of no service (Table 1). We based the categorization of services on the categories of the German Pension Insurance and adopted employer benefits and preparatory measures and differentiated vocational training benefits according to integration and qualification, as these are two different approaches to qualify for a job. One more category is for other services.

**Table 1:** Description of monthly states of VR service categories financed by the Federal German Pension Insurance in Germany and the state of no service

Service state	Explanation	Average length of service
Two-year vocational retraining	Transition to another suitable occupation through acquisition of a new professional qualification	24 months
One-year vocational retraining	Professional training and further education without acquisition of a new professional qualification	6 to 12 months
Integration service	Expansion and repetition of professional knowledge through time-limited practical work and job application training	6 to 9 months
Preparatory services	Preparation for qualifying services, tests to find a profession, and work testing	Up to 1 month
Employer benefits	Financial support for an employer	Up to 6 months
Other services	Change of workplace at the same employer or to another employer, support for mobility, support for rebuilding, technical working aids, temporary financial support for the applicant, work assistance, integration specialist service, sheltered workshop for people with disabilities, support for self-employment	Varying depending on type of service
No service	No documented service or services not considered due to incomplete data	Not applicable

The complexity of the services states decreases from top to bottom in the table. More complex services states were given priority in the consolidation of overlapping and parallel services

Two-year vocational retraining programs are common VR services in Germany to acquire a new job qualification while one-year vocational retraining programs provide further vocational training in addition to previous professional experience without acquiring a new professional qualification. In integration services participants receive support in applying for temporary internships in companies with the aim of the reentry into a stable employment. Persons with states of no service had in the month concerned no documented service or services that were not considered due to incomplete data.

To present VR services as sequences, we had to exclude services with missing information on the type, start date or end date; services starting or ending before January 2016 and identical services running in parallel. To have only one service per person and month, concurrent and overlapping states of services with different service types were purged, corresponding to the complexity of states of service categories (see Table 1). More complex services states were given priority in the consolidation of overlapping and parallel services. In Table 1, the most complex service state is at the top (two-year vocational retraining) and the least complex service state (no service) at the bottom.

In addition, the main service was identified for each person. We defined the main service as the service positioned highest in the hierarchy of VR services concerning the whole observation period. An integration service as a main service thus means that the person has an integration service as the highest service category in his or her sequence. This person may also have other services, such as preparatory services or employer benefits, but no one-year or two-year vocational retraining.

### Health, work ability, and sociodemographic data

For baseline health we assessed general health, physical functioning and self-reported diseases. General health was assessed with one item from the Copenhagen Psychosocial Questionnaire (COPSOQ) asking “If you evaluate the best conceivable state of health at 10 points and the worst at 0 points: How many points do you then give to your present state of health?” The item ranges from 0 to 10, higher values indicate better general health [26]. The COPSOQ is a well-established instrument to measure psychosocial stress at work and the German version shows good reliability [27].

We assessed physical functioning using the subscale physical functioning of the Short Form-36 (SF-36). The scale comprises 10 items that ask about restrictions in different daily tasks. The score ranges from 0 to 100 points, higher values indicate better physical functioning [28]. The German version of the instrument in reliable [29].

Self-reported diseases were assessed through the request to enter the current illnesses or injuries diagnosed by a doctor. The response options included cardiovascular diseases, cancer, skeletal, muscular and connective tissue diseases, mental/psychiatric diseases, nervous system diseases, respiratory diseases, diabetes, thyroid diseases or obesity, digestive diseases, urinary or reproductive diseases, allergies, infections, skin diseases and injuries and poisoning. We assessed the most common diseases in adults in Germany as proposed by the Robert Koch Institute [30].

Data on work ability were collected using the one item Work Ability Score (WAS): “Assume that your work ability at its best has a value of 10 points. How many points would you give your current work ability?”. The scale rates from 0 (“completely unable to work”) to 10 (“maximal work ability”) points [31]. Reliability of the WAS reached good results [32].

We assessed the self-reported risk of future work disability using a brief 3-item scale on the subjective prognosis of employability (SPE). The three items ask for the probability to continue in the current or in the last job until retirement age, the extent to which the current state of health permanently jeopardizes the work ability, and the intention to apply for a pension (0–3 points in total, higher values indicate a higher risk of future work disability) [33, 34]. The German version of the scale reached good reliability [35]. The self-reported risk of future work disability was grouped into people without (0–1 points) and with (2–3 points) risk of future work disability.

For sociodemographic data we assessed the educational level (low: up to 9 years in school or other graduation; medium: 10 to 12 years in school; high: 13 years in school). Age and gender were obtained from administrative data.

### Statistical analysis

Descriptive statistics characterized the full sample, frequencies of services, and clusters of services. Individual service sequences were analyzed and presented using the Sequence Analysis Tool for Stata [36]. Sequence analysis is an exploratory method to describe the whole process of defined states and their order in a fixed time period. An important step in sequence analysis is to determine similarities and differences between sequences by using distance measures. The distance measures can be used to describe groups of similar sequences and to compare different groups of sequences [37]. We used the optimal matching algorithm (OMA) to compare the individual sequences of VR services [38]. The algorithm identifies similar structures within the sequences that can be summarized to typical clusters of service sequences. The distance between any two sequences is understood as the cost of the cheapest set of edits (insertions, deletions, and substitutions) that will turn one sequence into the other [36]. We defined the costs of insertion and deletion (“indel costs”) at 1 and substitution costs at 2 so that substitutions are as expensive as one insertion and one deletion and are interchangeable in their use. A matrix of distances for all sequence combinations was generated. Sequence analysis is always a balance between reducing the variance of the sequences and preserving the sequential character of the data. Brzinsky-Fay proposes five steps to analyze sequences including the description and visualization of sequences, the comparison of sequences using distance measures and the identification of similar groups of sequences and using of the grouping variables for example in the description of the groups with additional variables or the use of the grouping variable as dependent or independent variables in regression models [39].

Cluster analyses were used to identify typical service sequences for individuals with integration services and two-year vocational reintegration as main services. We clustered only in those two groups, as these are the two most common main services and comprise more than half of our sample. We did not include the other main services that contain smaller subgroups in order to be able to describe typical and frequent sequences in our sample. The number of clusters was selected with the aim of describing distinguishable and meaningful groupings in terms of content. In addition, we calculated the Calinski-Harabasz index to find the statistical optimal number of clusters [40]. The highest index value indicates the best number of clusters. The index suggests two clusters for both subgroups. We decided to present three clusters in integration services as main services due to the clear difference in content.

Finally, we described the groups of individuals in the different clusters based on the sociodemographic and health-related characteristics. For the cluster description we excluded cases with missing data pairwise. Statistically significant differences between the clusters and the main services were calculated using Jonckheere trend test, chi-square-tests, analysis of variance and t-tests for independent samples. Effect sizes were measured using Eta squared (ƞ2; small effect ƞ2 ≥ 0.01, medium effect ƞ2 ≥ 0.06, large effect ƞ2 ≥ 0.14) for continuous and Cramers V (V; small effect V ≥ 0.1, medium effect V ≥ 0.3, large effect V ≥ 0.5) for categorial variables [41]. The calculations were performed in Stata SE 15 and IBM SPSS Statistics 22.

## Results

### Sample characteristics

A total of 7,008 individuals with approved VR were asked to participate in the study. 3,197 individuals completed the questionnaire, and 2,549 consented to the use of administrative data. Information on VR services was available for 1,918 participants. After deleting incomplete data, VR services could be presented as sequences for 1,652 individuals. In this sample, each person had some kind of VR service for at least one month within the observation period. 67.9% of the participants were female. The average age of the sample was 46 years (SD = 9.3). Participants reported poor general health and poor work ability. More than half of the sample (59.3%) had a self-reported high risk of future work disability. Every second person (55.4%) reported mental disorders (Table 2).

**Table 2:** Sociodemographic and health-related sample characteristics at the time of VR approval

	Total number	Number of observations	% or Mean (SD)
**Total**	1,652		
***Sociodemographics***			
Sex: female	1,652	1,121	67.9
Age	1,652		46.0 (9.3)
Educational level^a^	1,643		
Low		451	27.4
Medium		1,000	60.9
High		192	11.7
***Health***			
General health (0–10)	1,623		4.9 (2.0)
Physical functioning (0-100)	1,640		63.3 (24.5)
Mental disorders	1,652	915	55.4
***Work ability***			
Work ability score (0–10)	1,623		4.8 (2.4)
Risk of future work disability (0–3)	1,543		
No (0–1)		628	40.7
Yes (2–3)		915	59.3

^a^ Educational level: low (up to 9 years in school or other graduation), medium (10 to 12 years in school), high (13 years in school)

## Frequencies of services and main services

In total, 2,574 services were documented between January 1, 2016, and April 30, 2020. On average, every person had 1.3 different types of services (SD = 0.5) during this time. People had a minimum of one and a maximum of four different types of services in the sequence. 71.3% of the sample had only one service type, 25.4% had two, and 3.3% had at least three different types in the sequence. First services were most often integration services (36.6% of first services) and preparatory services (35.8% of first services).

Half of the sample started their first service between 92 days (3 months) and 192 days (6.5 months) after approval (interquartile range). On average the first service started 164 days (5.5 months) after approval. Individuals with preparatory services as main services started on average after 159 days (5 months) and individuals with employer benefits as the main service after 219 days (7 months). Service sequences were mainly between 74 days (2.5 months) and 272 days (9 months) long (interquartile range). People with only one type of service were on average longer in a service (182 days) than people with several different types of services (119 days).

Integration services were the most common main services in the sample (40.2%), followed by two-year vocational retraining (21.3%) and preparatory services (20.8%). Nearly half (45.9%) of two-year vocational retraining as a main service was initiated by a preparatory service, compared to only 13.0% of integration services. Employer benefits followed 8.5% of two-year vocational retraining and 15.9% of integration services (Table 3). The average duration of services was longest for two-year vocational retraining (257 days) and shortest for preparatory services (49 days).

While people with integration services as main services had on average 1.4 (SD = 0.6) services in their sequence with 29.1% who had two or more different services, individuals with two-year vocational retraining as a main service had on average 2.0 (SD = 1.0) services in their sequence, and nearly two thirds of sequences consisted of two or more different services (60.4%) (Table 3)

**Table 3:** Frequency and proportion of service types in sequences with different main services

	Number (%)	Number (%) One service type	Additional services to the main service				
**Total**	1,652 (100.0)	1,178 (71.3)					
**Main services**			**Number (%)** **One-year vocational retraining**	**Number (%)** **Integration service**	**Number (%)** **Preparatory services**	**Number (%)** **Employer benefits**	**Number (%)** **Other services**
**Two-year vocational retraining**	351 (21.2)	139 (39.6)	9 (2.6)	39 (11.1)	161 (45.9)	30 (8.5)	11 (3.1)
**One-year vocational retraining**	97 (5.9)	67 (69.1)		12 (12.4)	12 (12.4)	10 (10.3)	3 (3.1)
**Integration service**	664 (40.2)	471 (70.9)			86 (13.0)	99 (14.9)	27 (4.1)
**Preparatory services**	344 (20.8)	320 (93.0)				11 (3.2)	16 (4.7)
**Employer benefits**	65 (3.9)	64 (98.5)					1 (1.5)
**Other services**	131 (7.9)	117 (89.3)					

Additional services to the main service show services that occur in addition to the main service in the sequence. The percentages presented relate to the population of the corresponding main service

### Vocational rehabilitation service sequences

**Fig. 1:**
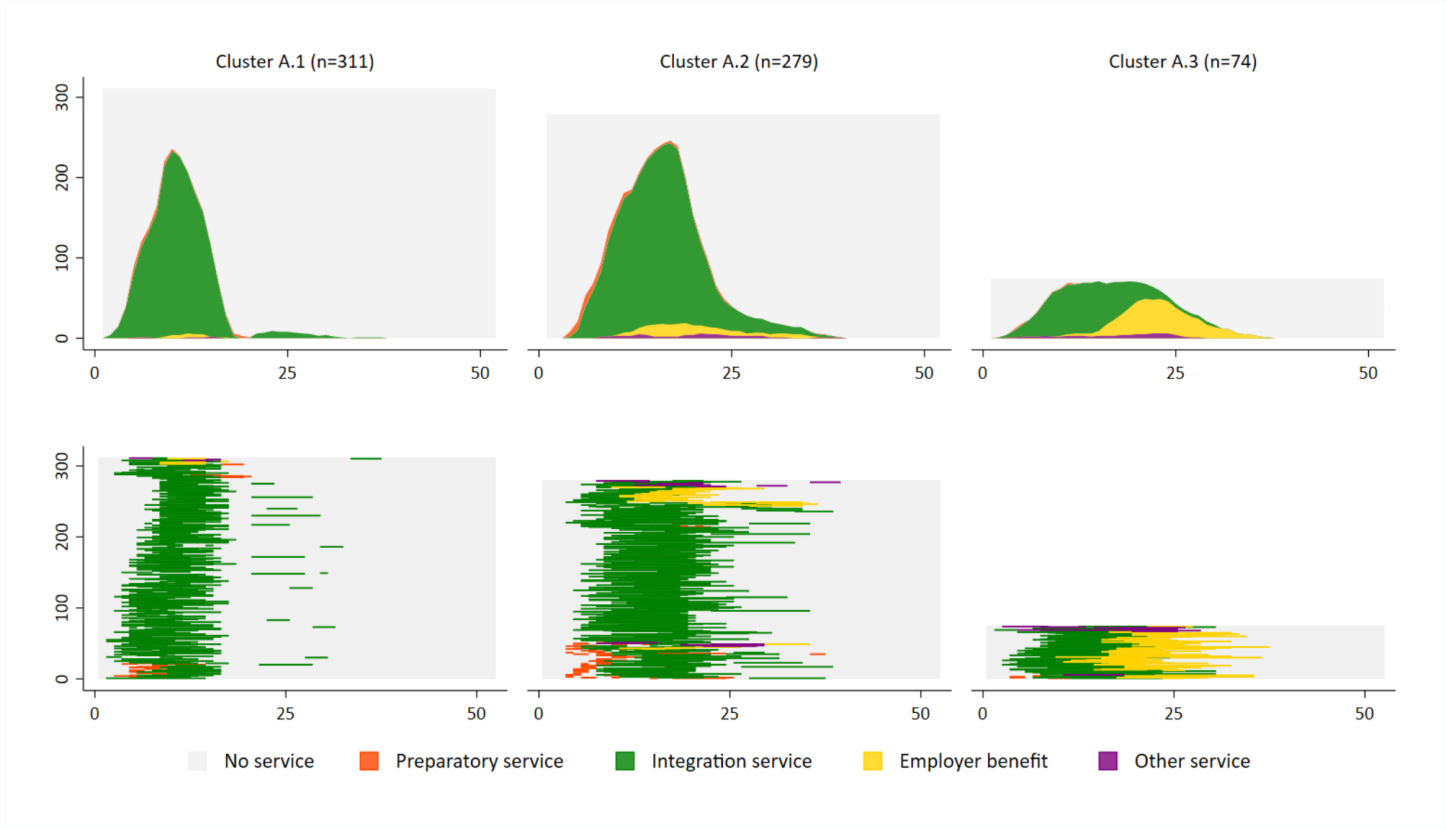
Typical VR sequences for people with integration services as main services; n = 664; Cluster A.1: Short integration services (n = 311); Cluster A.2: Integration services without employer benefits (n = 279); Cluster A.3: Integration services with employer benefits (n = 74); x-axis: Months 1 to 52; y-axis: Number of observations per cluster

Among the 664 people with integration service as a main service, three clusters were identified. Cluster A.1 consisted of 311 individuals (46.8%) with a short integration service that lasted on average 170 days. These short services were rarely initiated by preparatory services or followed by employer benefits. Cluster A.2 included 279 individuals (42.0%) with longer integration services (Mean: 264 days) followed in 32 cases (11.5%) by employer benefits. The proportion of preparatory services was highest in this group with 19.0% of cases. Cluster A.3 consisted of 74 individuals (11.1%) with integration services of whom nearly all (82.4%) were provided an employer benefit afterwards. Integration services were longest in this group, with a mean of 298 days (Fig. 1).

Individuals in shorter integration services (cluster A.1) reported worse general health, worse physical health, and more often a high risk of future work disability than individuals in longer integration services (clusters A.2 and A.3) (Table 4).

Within the group of individuals with two-year vocational retraining as a main service, two clusters were identified.

Cluster B.1 consisted of 187 individuals (53.3%) with shorter vocational retraining services that lasted on average 196 days (6.5 months). About one third of the sequences (32.1%) in this cluster started with preparatory services, and 11.8% had integration services before two-year vocational retraining. There were nearly no employer benefits at the end of the sequences (3.7%).

**Fig. 2:**
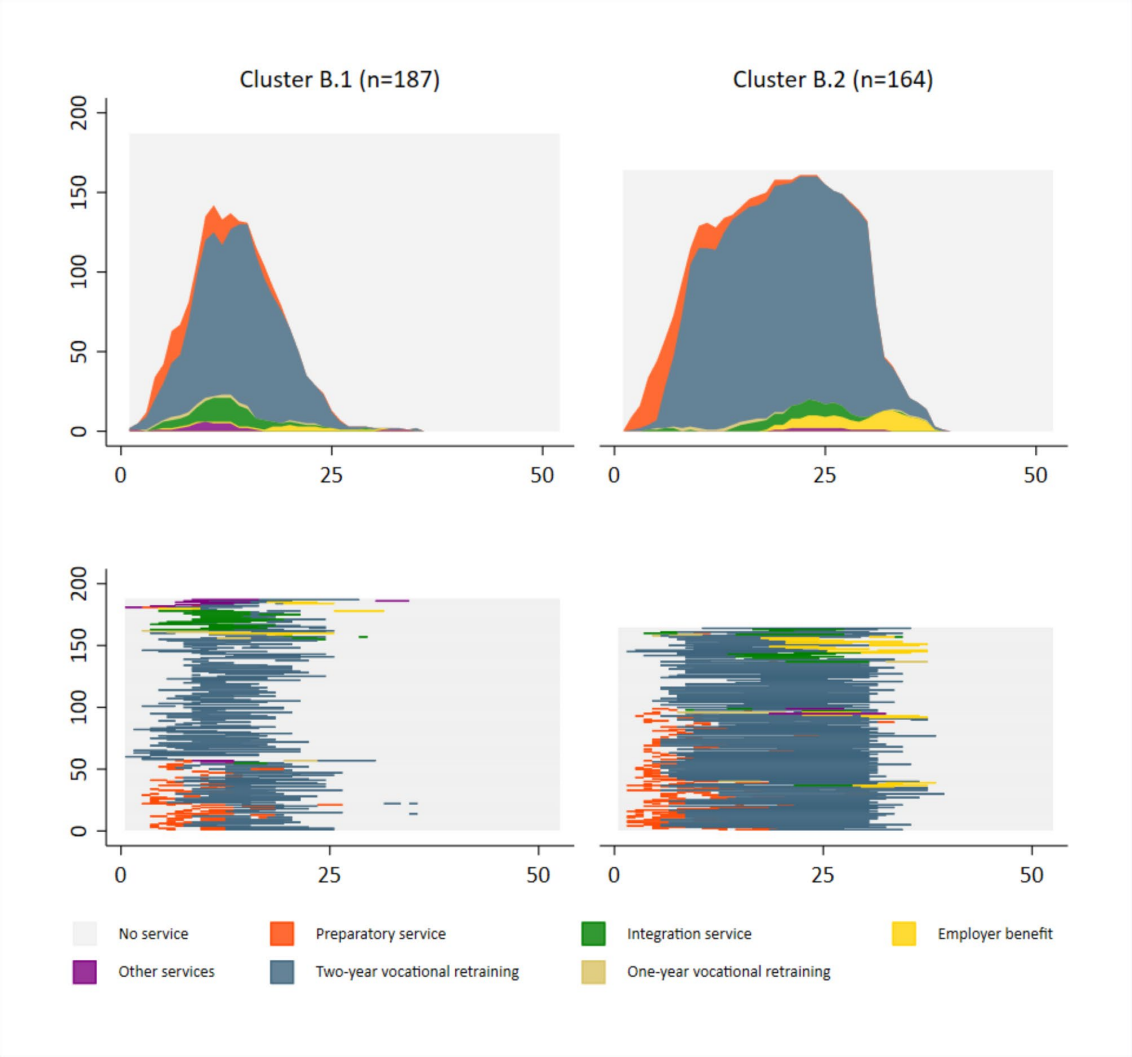
Typical VR sequences for people with two-year vocational retraining as main service (n = 351); Cluster B.1: Shorter two-year vocational retraining services (n = 187); Cluster B.2: Longer two-year vocational retraining services (n = 164); x-axis: Months 1 to 52; y-axis: Number of observations per cluster

Cluster B.2 included 164 individuals (46.7%) with longer two-year vocational retraining that lasted on average 549 days (about 18 months). Main services in this cluster were in more than every second person preceded by preparatory services (61.6%), and 14.0% of services were followed by employer benefits. About as many people as in cluster B.1 had integration services before two-year vocational retraining (10.4%) (Fig. 2).

Individuals in longer services, both two-year vocational retraining and integration services, reported better physical health (for Clusters in integration services ƞ^2^=0.023) and better work ability, were less likely to have a high risk of future work disability, and were younger than individuals in shorter services (for Clusters in integration services ƞ^2^=0.012; for Clusters in two-year vocational retraining ƞ^2^=0.067). When comparing the two main types of services, individuals in integration services reported poorer general health (ƞ^2^=0.027), worse work ability (ƞ^2^=0.029), more often a high risk of future work disability (V = 0.151), more often mental illness (V = 0.130) and were older (ƞ^2^=0.091) than individuals in two-year vocational retraining (Table 4).

**Table 4:** Sociodemographic and health-related characteristics of clusters with integration services and two-year vocational retraining as main services

	Integration services as main service				Two-year vocational retraining as main service		
	Total	Cluster A.1	Cluster A.2	Cluster A.3	Total	Cluster B.1	Cluster B.2
**Total number**	664	311	279	74	351	187	164
***Sociodemographics***							
Sex: female, n (%)	460 (69.3)	216 (69.5)	190 (68.1)	54 (73.0)	238 (67.8)	131 (70.1)	107 (65.2)
Age, Mean (SD)	49.0 (7.8)**	49.4 (7.7)*	48.1 (8.0)*	50.6 (7.5)*	43.7 (9.2)**	45.9 (9.3)*	41.2 (8.5)*
Educational level^a^, n (%)							
Low	186 (28.2)	90 (29.3)	83 (29.9)	13 (17.6)	92 (26.4)	61 (33.0)*	31 (18.9)*
Medium	399 (60.5)	186 (60.6)	161 (57.9)	52 (70.3)	219 (62.8)	104 (56.2)*	115 (70.1)*
High	74 (11.2)	31 (10.1)	34 (12.2)	9 (12.2)	38 (10.9)	20 (10.8)*	18 (11.0)*
***Health***							
General health (0–10), Mean (SD)	4.6 (2.0)**	4.5 (2.0)	4.6 (1.9)	5.1 (1.8)	5.3 (1.8)**	5.3 (1.9)	5.2 (1.8)
Physical functioning (0-100), Mean (SD)	61.7 (24.8)	59.7 (24.9)*	61.3 (25.2)*	72.0 (20.4)*	64.7 (23.3)	63.0 (23.7)	66.7 (22.7)
Self-reported mental disorders, n (%)	391 (58.9)**	173 (55.6)	172 (61.6)	46 (62.2)	159 (45.3)**	81 (43.3)	78 (47.6)
***Work ability***							
Work ability score (0–10), Mean (SD)	4.5 (2.4)**	4.5 (2.5)	4.3 (2.3)	4.9 (2.3)	5.3 (2.4)**	5.1 (2.4)	5.6 (2.3)
Risk of future work disability, n (%)							
No (0–1)	218 (35.6)**	91 (32.2)	98 (38.0)	29 (40.8)	171 (51.2)**	85 (48.0)	86 (54.8)
Yes (2–3)	394 (64.4)**	192 (67.8)	160 (62.0)	42 (59.2)	163 (48.8)**	92 (52.0)	71 (45.2)

Sample size varies per row due to missing data; missing data in integration services as main services: education (0.8%), general health (1.8%), physical functioning (0.8%), work ability (1.5%), risk of future work disability (7.8%); missing data in two-year vocational retraining as main service: education (0.6%), general health (2.0%), physical functioning (0.6%), work ability (2.3%), risk of future work disability (4.8%); SD = Standard deviation; ^a^ Educational level: low (up to 9 years in school or other graduation), medium (10 to 12 years in school), high (13 years in school); * Statistically significant differences between the clusters within integration services and within two-year vocational retraining using Jonckheere trend test, chi-square-tests, analysis of variance and t-tests for independent samples (all tests *p* < 0.05); ** Statistically significant differences between the main services using Jonckheere trend test, chi-square-tests and t-tests for independent samples (all tests *p* < 0.05)

## Discussion

Our analyses described sequences of VR services in Germany for a period of almost four years after approval. In most of the observed sequences (40%), an integration service was the main service. Integration services are generally categorized as educational services and account for approximately 17% of completed services at the Federal German Pension Insurance [14].

Multiple distinct services, that is, sequences of services, were more likely among individuals who had more complex services as their main services. Preparatory services and employer benefits were found more frequently among those with two-year vocational retraining than integration services. This is consistent with results from a previous analysis of typical sequences of VR in Germany [18]. Complex and expensive services are more frequently complemented by other services, possibly to maximize the impact of complex services. A study shows that individuals with longer periods of services had a more stable return to work compared to individuals with a shorter time of services [42]. It is likely that longer periods of services are more complex services and probably more often sequences of different services.

In addition to the advantage of complex services in terms of the outcome we could observe that younger and healthier individuals were more likely to receive complex services. Individuals in two-year vocational retraining were about six years younger (43.7 years) than individuals in integration services (49.0 years). Another study that examined the outcomes of one-year and two-year vocational retraining services found an even lower mean age for those in two-year vocational retraining services of about 38 years [43].

It is possible that we have a situation here, in which people with better qualifications are preferred, as they are more likely to receive more effective services that may lead to a successful return to work. There is an assumption, that more highly educated people have an advantage in the negotiation process with rehabilitation counsellors when selecting VR services [44].

When it comes to the allocation of services, the initial occupation before entering the VR services plays also a role. If occupational activities are less in demand due to for example digitalization, reintegration into this occupational field could be less sustainable. This could also apply to traditional skilled trades. As a high regional unemployment reduces the chances of return to work after vocational training services [45], those people would be more likely to receive a vocational retraining to pursue a new profession than a service to reintegrate them into their previous profession.

Mental health problems occurred more often in people with integration services than with two-year vocational retraining. This may indicate that individuals with mental illness were less likely to receive complex services. Reasons for this could be that counselors in service selection were less likely to consider people with mental illness to be capable of handling the educational complexity of retraining or to have the resilience or endurance required to complete a two-year vocational retraining program. Furthermore, the fact that there were more people with mental impairments in the shortened services suggests that poor mental health could be a reason for dropping out of services. Ending an educational service early, before the regular end date, increased the risk of no return to work after VR [46]. Furthermore, medical reasons were reported as the most common cause of early drop out of services [43]. Mental health problems are particularly relevant in this context because having mental health problems is a risk factor for work disability and unemployment after VR [21]. There may be a need for further support for this target group that could integrate mental healthcare with VR services [47].

We see a proportion of non-linear VR sequences, i.e. sequences that cannot be mapped along typical service sequences like preparation, vocational retraining and employer benefits, that do not correspond to the typical duration of services or repeated services of one type. On the one hand, this is not surprising, as vocational rehabilitation sequences can extend over a longer period of several months or even years and the probability of particular health or family events which make it necessary to terminate or interrupt the VR process is high. On the other hand, it seems that the intended impact of services and service sequences may not be fully utilized and that certain subgroups may have a need for individual support. The Federal German Pension Insurance reports that in 2020 around 20% of educational services (including vocational retraining and integration services) are discontinued, often for health reasons [15]. We need to monitor, what happens to people with non-linear VR sequences or aborted services.

In addition to health, low education appears to be another factor limiting the duration of services below the usual length. We observed more persons with low education in shortened two-year vocational retraining and in shortened integration services than in longer services. It could be that individuals with lower educational levels are more likely to enter a job for financial reasons early before they complete the service. A German qualitative study on discontinuation of VR services identified financial concerns as one possible reason for dropout [48].

The fact that age, health, and education appear to be relevant factors not only for the assignment to services but also for the duration of services supports the need to monitor sequences of individuals with multiple health problems when entering the VR process. It seems necessary to integrate more individual assistance into the process of VR. Vocational rehabilitation facilities sometimes offer services such as vocational retraining that are aimed at specific subgroups, e.g. people with addiction problems, mental impairments, people with autism, hearing impairments or neurological disorders. The main challenges here are the regionally limited range of services and the communication of these services. Many services are often unknown to those affected and also to those providing advice. Information about such offers could be disseminated more widely, possibly also through more intensive training of rehabilitation counsellors.

In order to evaluate the displayed sequences, we need to analyze data on occupations after the VR services. We need further research that assesses the different effects of the sequences against the background of the initial occupations and target occupations. We do not yet know when and in which occupations people return after various sequences. There is also need to know what happens in the long term with non-linear VR sequences and what kind of support is necessary for people affected.

### Strengths and limitations of our study

The results must be interpreted in the light of the following limitations. First, in order to use the data in sequence analysis and to be able to present the services as sequences, the data had to be categorized into service categories and to be edited as mentioned above. This reduced the complexity of service sequences. We were not able to consider parallel services or represent service interruptions or endings for various reasons (health reasons or return to work) or present services without information on beginning and ending. It also reduced the number of cases whose sequences could be represented and included in this analysis. A consequence is also that our sample is not representative of all people receiving VR services. Since we reduced the clustering to the two most frequent main services integration services and two-year vocational retraining, we cannot map any sequences with other main services. This would probably reveal further typical and possibly non-linear sequences, e.g. for people who have only preparatory services as their main services. At this point, we need larger samples in order to be able to map more sequences. Second, regardless of the preparation of the data, the administrative data were partly incomplete. This may have led to some misclassifications and miscoding. Third, our data represent only services directed by the Federal German Pension Insurance, although there are other providers of VR services in Germany. Sociodemographic characteristics of insured people differ from other providers in age, gender, and received services [14].

In contrast, the analysis has several strengths. First, we were able to plot service sequences over their entire length for a large sample for the first time. So far, little is known about sequences of VR services in Germany. The results provide an insight into typical service sequences within the most common educational services and reveal the actual duration of these services. Second, we considered a long period of four years and could describe typical sequences of VR services for a sample with different health impairments. Third, we used administrative data to describe the services and linked them to self-reported data on health and work ability.

## Conclusions

We conclude that multiple different services were more common among individuals with more complex services like two-year vocational retraining. Furthermore, the use of two-year vocational retraining as a complex and expensive service was influenced by health, age, risk of future work disability, and education. Also, the duration of the services was associated with health-related variables, age, and education. We noticed that in particular individuals with mental impairments received shorter services and thus may need further support in their process of vocational reintegration.

The fact that age, health, and education appear to be relevant factors not only for the assignment to services but also for the duration of services supports the need to monitor sequences of individuals with multiple health problems when entering the VR process.

## Data Availability

The datasets generated and/or analysed during the current study are not publicly available due to privacy or ethical restrictions but are available from the corresponding author on reasonable request.

## Abbreviations

VR: vocational rehabilitation
